# Resource allocation for environmental health services in healthcare facilities: A qualitative case study from Niger

**DOI:** 10.1371/journal.pwat.0000330

**Published:** 2025-02-28

**Authors:** Silvia Landa, Elisha Y. Sanoussi, Ezechiel Mahamane, Kairou Oudou Bilo Mahamadou, Aaron Salzberg, Darcy M. Anderson

**Affiliations:** 1 The Water Institute at UNC, Gillings School of Global Public Health, The University of North Carolina at Chapel Hill, Chapel Hill, North Carolina, United States of America,; 2 Department of Bioengineering and Therapeutic Sciences, University of California San Francisco/University of California, Berkeley, California, United States of America,; 3 World Vision Niger, Nouveau Marche, Niamey, Niger

## Abstract

Water, sanitation, hygiene, waste management, and other environmental health services are critical for safe health systems, but global access is lacking. Adequate financing is a key barrier, and understanding resource allocation can help identify solutions in resource-limited contexts. We conducted a qualitative case examining resource allocation in rural Niger. Our objectives were to understand resource allocation processes, key actors and their roles, and contextual factors that influenced resource allocation. We interviewed thirty-three healthcare workers, community leaders, and government officials. We found that resource allocation followed formal and informal processes. Formal processes encompassed annual budgets and monthly supplies through government channels, while informal processes depended on healthcare workers’ out-of-pocket expenses, unpaid labor, in-kind community support, healthcare facility revenue, and contributions from non-governmental and United Nations agencies, and the diaspora. Informal resource allocation was critical to fill the gap when formal processes were slow or insufficient. Resource allocation was highly decentralized with minimal influence of national policies and legal frameworks at the local level. Key contextual factors influencing resource allocation included politicization of budgets at the commune level, sometimes leading to inefficiency or inequity. We observed that healthcare facility actors who were most knowledgeable of needs often held the least decision-making power. We concluded that informal processes were complementary to formal processes, not conflicting. In contexts where government funding is severely limited, informal mechanisms may be the only viable short-term option to ensure the availability of services, demonstrating greater flexibility and adaptability. However, ultimately informal processes are an interim solution that should be explored to ensure service delivery without undermining long-term government systems strengthening. We recommend that funders commit to long-term initiatives promoting local government democratic decision-making, account for local actors’ capacities and incentives, and acknowledge dynamic formal and informal resource allocations to optimize investments and trade-offs.

## Introduction

Environmental health services (i.e., the suite of infrastructure and activities that create a built environment that enables safe and quality healthcare delivery, including water, sanitation, hygiene, waste management, cleaning, and energy [1]) are essential for safe healthcare delivery. Adequate environmental health services reduce healthcare acquired infections, slow development of antimicrobial resistance [2–5] and improve the quality of care and patient experiences [6–8]. Despite their importance, significant global shortfalls exist in access, particularly in low- and middle-income countries. According to monitoring data from the World Health Organization (WHO) and United Nations Children’s Fund (UNICEF), 49% of healthcare facilities globally lack basic hygiene services, and 22% lack basic water services. In the least developed countries, deficiencies are even more prevalent; 68% lack basic hygiene, 66% lack basic waste management, 47% lack basic water services, and 79% lack basic sanitation [9].

Limited budget and human resources for environmental health services within health systems are a barrier to strengthening coverage and access [10–12]. Although 66% of country es have cost estimates for water, sanitation, and hygiene (WASH) plans for healthcare facilities, only 3% have adequate human and financial resources for implementation [13]. Whether or not sufficient resources are available for environmental health services depends on the total resources available within the health system but also decisions about resource allocation. Resource allocation is the process of distributing finite resources within the health system for specific purposes, making choices between competing needs and priorities [14]. In contexts where the total resources available for health system financing are substantially below what is needed to meet basic population health needs, environmental health services are often deprioritized compared to other expenses with more directly observable, immediate health impacts [15].

Resource allocation for environmental health services in healthcare facilities is complex, particularly within low-resource settings where funding may come from a mixture of national resources, official development aid, non-governmental organizations (NGOs), and other sources. The mixture of funding may also change over a project’s lifespan, for example with official development aid or NGOs contributing to up-front capital investments but not longer-term operations and maintenance [16]. Stakeholders, including healthcare providers and administrators, may also have different priorities when making resource allocation decisions [17–19].

Understanding resource allocation for environmental health services within health systems is important for addressing financial barriers. Successful resource allocation requires consensus and collaboration across stakeholders, such as workers, administrators, NGOs, and the community [20–22]. A clear understanding of the current practices helps create realistic strategies for resource-limited contexts. When resource allocation systems are understood, they can be more effectively targeted for change. For example, one case study in Nepal reported an advocacy campaign that identified and targeted key financial decision makers within local government, resulting in increased financing to operate and maintain environmental infrastructure in healthcare facilities [23].

Yet there is little research specifically on resource allocation. A majority of evidence on financing of environmental health services in healthcare facilities focuses on calculating the costs of service delivery—both providing country-specific costs estimates and tools for cost data collection [15,18,23–25]. There are a small number of case studies that examine resource allocation at the national level for specific policies—for example environmental health policy in healthcare facilities in Malawi [21] and waste management policy in Saudi Arabian healthcare facilities [22]. However, there is little in-depth study of how local actors manage resource allocation in resource-limited settings.

We addressed this research gap through a qualitative case study in rural Niger. We examined how local actors navigated resource allocation in a resource-limited context. We aimed to (1) document the processes for resource allocation, (2) describe the key actors and their roles in resource allocation and (3) explore the contextual factors that influenced resource allocation. These findings can inform more effective and context-sensitive interventions, ensuring that policies and programs align with the realities of local decision-making and resource allocation.

## Methods

### Study overview

This study was conducted in partnership with the NGO World Vision. In 2022, World Vision began delivering a program to improve environmental health services in 34 rural healthcare facilities in rural Niger. The program comprised infrastructure construction or rehabilitation of water sources, water towers, and solar pumping; sanitation blocks including toilets and showers; and incinerators for waste management. Healthcare facilities were provided supplies to operate and maintain this infrastructure for approximately six months, with the intent that local governments and communities would assume responsibility for management after the program’s end. Approximately six months of supplies were also provided to support cleaning and hand hygiene (e.g., soap, bleach, mops).

This study was conducted to understand local systems for resource allocation that would influence the financing and sustainability of these improvements after formal program support ended. As such, we examined resource allocation for environmental health services targeted by the program: water, sanitation, hygiene, cleaning, and waste management. This scope is aligned with what is monitored for environmental health services in healthcare facilities under Sustainable Development Goal 6 for water and sanitation in healthcare facilities [24].

### Conceptual framework

We used the policy triangle framework [25] to understand how local actors navigate the resource allocation process and the contextual factors influencing their decisions. The policy triangle framework was developed by Walt and Gilson [25] for health policy analysis, to explain the factors that drive successful implementation of a health policy. It has been widely used in the health sector for policy analysis [26]—including environmental health policies [22,27–29]— and more broadly to understand government decisions and propose potential changes [26].

For this research, we drew on three relevant constructs from the policy triangle framework that we hypothesized influenced the resource allocation—process, actors, and context—corresponding to our three research objectives. Definitions are provided in Table 1.

### Health policy context in Niger

Niger’s administrative structure comprises three sub-national levels: regions, departments, and communes. Communes or town halls—led by elected mayors and councils—are crucial for local decision-making and have strong financial autonomy. Departments—led by prefects—act as intermediaries between communes and regions and ensure the implementation of national policies. Regions construct, maintain, and manage regional hospitals, which serve as referral hospitals, manage smaller healthcare facilities, and oversee policy implementation and monitoring. Governmental technical agencies are primarily located at the departmental level and staffed by bureaucrats from diverse sectors [30,31].

The health sector employs a distinctive arrangement where health districts operate comparably to departments regarding their roles and influence, though geographic boundaries of health districts may not overlap with those of departments. Primary care in rural areas is predominantly provided by integrated health centers, which are supported by several smaller health posts. These integrated health centers manage the health posts within their catchment areas and liaise with district hospitals and commune governments to manage the logistics of budgeting and procurement, including for health posts in their area [30,31]. All healthcare facilities in our study were integrated health centers.

Community committees—notably COGES (short for *comités de gestion* or management committees) and COSAN (short for *comités de santé or* health committees)—support health workers and healthcare facilities services. COGES manages cost recovery through service fees and pharmaceutical sales, which funds essential operations like pharmacy restocking and minor infrastructure repairs. Funds also cover staff salaries and bonuses, with additional support from the town hall [32–34]. COSAN promotes health awareness [34,35]. Both COGES and COSAN committee members are community residents; COGES includes additional healthcare facility representatives.

### Participants and sampling

This study was conducted in the Dosso and Maradi regions of Niger in communes receiving the World Vision program. Program healthcare facilities were selected based on need (lack of access to environmental health services at baseline), convenience (proximity to regional headquarters and accessibility), and security considerations. Characteristics of program healthcare facilities are presented in Table 2.

Our interview sample was drawn from healthcare facility administrators (e.g., directors, financial officers), COGES members at program healthcare facilities, and government bureaucrats and elected officials responsible for funding and regulating environmental health services at the commune and department levels in the program area. To recruit healthcare facility administrators and COGES members, we asked the directors of each healthcare facility to identify individuals in charge of environmental health, supervision, procurement, and management. We attempted to recruit one participant for every healthcare facility in the program (n = 34], though at some a knowledgeable participant was not available during the study visit. To recruit government stakeholders, World Vision program staff developed a shortlist of potential participants knowledgeable about environmental health services at the commune and department levels, based on a briefing about the study objectives, which was discussed and finalized with the research team.

### Data collection

We conducted qualitative interviews from 7^th^ – 28^th^ October 2022. We developed two distinct interview guides, one for healthcare facility level participants (administrators and COGES members) and one for government officials and bureaucrats. The healthcare facility guide focused on identifying specific challenges related to environmental health (e.g., infrastructure failures, shortages), investigating their root causes, and exploring the solutions employed and the contextual factors influencing these solutions, with particular attention to resource allocation issues such as inadequate funding and supply chain challenges. The government guide explored the daily management of environmental health services, strategies for ensuring resource availability, methods for obtaining additional support at healthcare facilities, and the determinants of these practices.

An interviewer and a note-taker conducted each interview in French, Hausa, or Zarma. Each interview lasted approximately one hour. For participants who consented, interviews were audio-recorded, transcribed in French, and translated into English for analysis. Where participants did not consent to audio-recording, we analyzed notes taken during the interview.

### Analysis

Three authors collaborated on the analysis. Two authors (SL and DA) developed an initial codebook with *a priori* codes from the Policy Triangle Framework (i.e., processes, actors, context, as defined in Table 1). Our results were organized and are presented under these domains. For the process domain, we separated processes into formal (i.e., resource allocation through official government mechanisms) and informal (i.e., resource allocation outside official government mechanisms). For the context domain, we created additional *a priori* sub-codes (political, legal, economic, sociocultural, epidemiological, ethical, and geographical) based on the Context and Implementation of Complex Interventions framework [36].

The lead author (SL) then applied the codebook to analyze a subset of six interviews. We convened to review the results and to refine and adjust the codes, incorporating *a posteriori* codes for emerging themes from the data until we reached a consensus. The lead author then coded the entire dataset using the final codebook. After coding, three authors (SL, ES, DA) debriefed the coded transcripts before drafting the manuscript. We provided the complete codebook in S1 Table.

### Ethics

This study was ruled as not human subjects research by the Institutional Review Board at the University of North Carolina at Chapel Hill and received local approval from the Niger Ministry of Health. We obtained permission from the director of each healthcare facility, and all interview participants provided written informed consent.

### Results

Our sample comprised 33 participants (Table 3). Among them, 11 were representatives of local governments, while the remainder consisted of healthcare facility personnel, COGES members, and community leaders involved in allocating resources for environmental health services. The average tenure of these participants in their respective roles was seven years. Additional details are provided in S1 Data.

### Resource allocation process

We identified formal and informal mechanisms for the resource allocation process. The formal process included government procedures for allocating monetary resources and government-paid personnel, usually following the government timeline. The informal process consisted of non-governmental budget sources and unpaid labor, like community volunteerism and civil society funding, free from the strict government timeline.

#### Formal.

**Fig 1.**
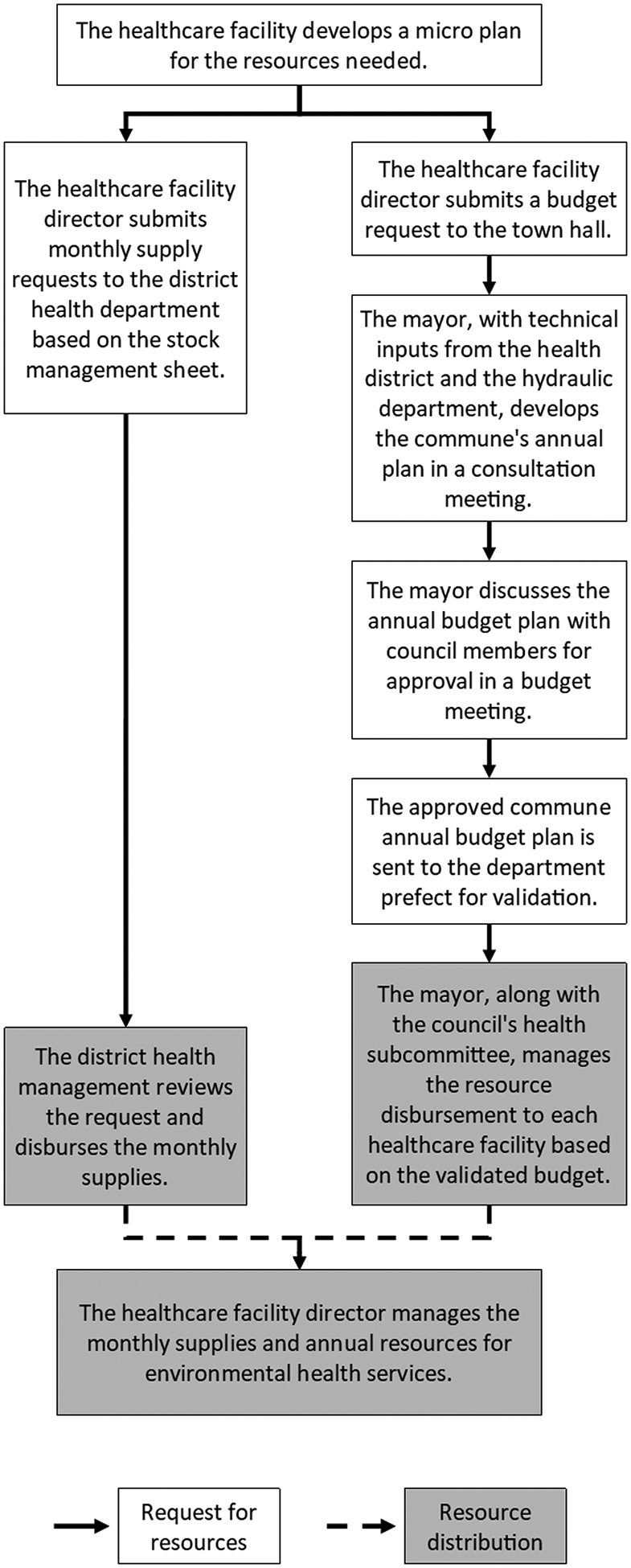
Formal resource allocation mechanism for environmental health services in rural Niger. https://doi.org/10.1371/journal.pwat.0000330.g001

Healthcare facilities primarily relied on two formal resource allocation mechanisms: annual budgets and monthly supply requests. These mechanisms were guided by a multi-year planning process that occurred within health districts, departments, and regions. Fig 1 depicts formal resource allocation processes at the commune level, which is started at the integrated health center level and includes all health posts in its catchment area. For simplicity, we did not illustrate the iterative process required for potential modifications or revisions of the budget across levels, for example if the budget submission for a given step were not approved. These revision processes are described narratively in subsequent sections.

Multi-year plans guided resource allocations at the highest level. Health districts and regional health authorities had five-year plans for directing annual development, for example by outlining goals and priorities. Multi-year and micro-plans addressed all health needs and were not specific to environmental health services. Departments also had multi-year plans, which were separate from health district plans. These plans overlapped with environmental health services within healthcare facilities for larger infrastructure investment projects for water and sanitation. For example, the hydraulics and sanitation sector developed a three-year plan detailing water and sanitation infrastructure conditions and needs throughout the department, including healthcare facilities.

Each year there is also our action plan that must be followed, and this year these are the actions that must be taken to implement it.… There is a financial development plan, which is for five years. We have to develop it for all the activities, all the actions brought within the level of the district. We program them for five years.

– District communications officer

Integrated health centers developed micro-plans to guide the flow of resource requests through various channels, ensuring the provision of all services at the healthcare facility level, including those at the health post within their catchment area. One key formal resource allocation channel was annual budget requests. Healthcare facility directors submitted annual budget requests to the commune town hall. The mayor then formulated the commune’s annual budget plan, incorporating technical inputs from health and hydraulic departments. Commune annual budget plans addressed concerns from local levels—including those from health and other sectors—which were gathered through a consultation meeting. After discussing the budget plan with council members and receiving approval, the mayor forwarded the annual budget to the department-level prefect for validation. The budgets were organized under specific headings to cater to different needs such as health and education. In collaboration with the council’s health subcommittee, the mayor oversaw the disbursement of resources to each healthcare facility based on the approved and validated budget. This process often involved iteration:

When we finish [the budget plan], we enter it. We send it to [the prefect]. He will study it, what is wrong. He will tell us. We correct it, and we give it to him again

– Deputy mayor

Annual budget allocations from the commune to healthcare facilities considered the number of health posts each integrated health center managed and the population size served. Environmental health services were not specifically earmarked within budgets. Healthcare facilities’ micro-plans offered general headings of the healthcare budget, with detailed allocations left to the facility director’s discretion. The director of each healthcare facility managed the monthly supplies and annual resources for environmental health services. Healthcare facilities could request budget adjustments from the town hall several times. However, after the approval of the annual budget, the mayor and council members could only reshuffle the budget once. This reshuffling, which did not guarantee an increase, typically involved reallocating funds from other budget headings, such as education to health, based on requests, technical inputs, and council approval. Participants often considered these formal mechanisms to be slow and insufficient to fully meet the demands for environmental health service supplies and infrastructure.

Healthcare facilities also requested monthly supplies for consumable products, such as soap, masks, gloves, brooms, and pharmaceuticals from the health district, using stock management sheets to check availability. However, these requests sometimes faced delays of up to two months. One facility preceptor described slow and unpredictable delivery of supplies:

They sometimes take time before delivering to us, so we say to each other that it’s been a while they haven’t come, and then suddenly we see them coming. [When orders are late,] we buy with our own funds, since we need supplies above all else.

– Facility preceptor

Health districts sometimes supplied healthcare facilities without specific requests, based on healthcare facilities’ size and resource availability at the health district. Besides sending monthly request forms, healthcare facilities could also make in-person requests at health district training sessions.

The amount of funding each health facility should receive … Always, it depends on the size [of the healthcare facility]

– Deputy Mayor

Monthly supply requests went to the health district for non-emergency situations. During emergencies like the COVID-19 pandemic or other epidemics, the town hall supplied consumable products.

#### Informal.

Informal resource allocation mechanisms were used to cope with insufficient resources received through formal processes. We identified four mechanisms for informal resource allocation: resources from civil society organizations, UN agencies, and diaspora; community voluntary support; facility revenues; and healthcare workers’ funds and unpaid labor. For large expenditures, healthcare facilities typically received resource allocations from NGOs, UN agencies, and the diaspora. For minor expenditures, they obtained community volunteer support (financial or in-kind donations such as labor) and facility revenues. Healthcare workers used their own money and unpaid labor as a last resort in severe financial shortages.

Healthcare facility stakeholders were permitted to request assistance directly from NGOs, while informing health district and commune stakeholders. Coordination for environmental health services with NGOs and UN agencies occurred at the health district and commune levels. NGOs distributed resources directly to healthcare facilities through intermediaries from health district or commune governments, or by hiring private contractors (e.g., to construct infrastructure). NGO funding was typically associated with a specific project (e.g., infrastructure construction and a limited period of operations and maintenance). However, facilities would appeal to NGOs for additional support beyond defined projects. One health district official described the process of communicating with NGOs:

To get funding, you have to draw up terms of reference, and we send them to the partners…. [NGOs] themselves, they can come to us to tell us, that, ‘here they are.’ Or we do an interview to tell them what we need. We tell them everything, and then they will see what they can help us with.

-District communications officer

We identified two sources of facility revenues used to fund environmental health services: the sale of water to villagers and healthcare service fees. This system favored facilities with operational water towers, enabling them to enhance their revenue. Legitimizing the increased service fee involved a consensus among key stakeholders, including the chief of COGES, the head of the healthcare facility, the village head, and the mayor. Revenue from water sales and additional service fees was typically submitted directly to the town hall. Funds were then disbursed to healthcare facilities as needed based on the availability of funds and the severity of the need. However, in urgent cases, the revenue collected at the healthcare facility could be used directly with authorization from the mayor.

All the money [from selling water] is brought to the town hall. We collect it every month to pay to the town hall. [When there is a breakdown], we must call them.

- Facility preceptor

Healthcare facilities received support from villagers, predominantly coordinated by various community committees, such as the WASH committee, COSAN, and COGES. The presence and activities of these committees varied across locations. When present, WASH committees led environmental health-related activities. Without a WASH committee, COSAN and COGES spearheaded community mobilization efforts, such as organizing villagers to clean healthcare facilities. COGES managed efforts for activities requiring modest funding, such as securing local labor at a reduced cost. Additionally, COGES and healthcare workers attempted to solicit donations from the villagers, such as water, soap, and small amounts of money, although these efforts did not uniformly meet with success.

Well, the role of the community is to help us make the service work well. During the rainy season the rain removed the roof… We asked for help from the fee collector, who went to see the committee [COGES]… because they even had a woman who came for the delivery. They quickly repaired the roof, and we started our operation.

- Nurse

When formal and other informal resource allocation processes failed, healthcare workers would often cover supply shortages with personal purchases and perform extra duties without pay during resource scarcities. Examples of extra unpaid duties included traveling long distances to fetch water when water sources were broken down. Typically, out of pocket expenses were for small consumable products, such as gloves, soap, mops, and brooms, and were most commonly made by senior staff (e.g., the facility director) and distributed across the healthcare facility as needed. One facility director described the process of receiving donations of supplies and using different funding sources when donations ran out:

Soaps, bleach… we can have donations. But when this is over, we must deduct from cost recovery funds to get paid. Or there are times that the healthcare facility chief takes it out of his pocket for missing things.

– Healthcare facility deputy director

### Actors

We categorized actors into government and non-government groups. Government actors consisted of individuals operating within the governmental system, including healthcare workers, health district bureaucrats, and elected officials (e.g., mayors and council members at the municipality level). Non-government actors were entities external to the government system, including villagers, village committees, and private individuals outside the village (e.g., the diaspora).

The relationships among actors were complex yet flexible, allowing for formal and informal resource allocation mechanisms. Governmental actors faced limitations imposed by their designated duties and levels of authority. Within the formal framework, each governmental entity operated strictly within its assigned jurisdiction, relying on higher authorities’ approval. Conversely, in informal resource allocation, relationships among actors were more flexible, eliminating the need for upper-level government approval, although coordination was expected. For instance, healthcare workers could directly engage with NGOs; however, when NGOs intended to allocate resources, they typically notified or coordinated with the commune government. COGES often directly contacted the mayor but kept the healthcare director informed.

#### Government actors.

The healthcare facility workers responsible for distributing resources for environmental health services were typically the tax collector, treasurer, and facility director. The tax collectors collected payments for healthcare services and water sales, and treasurers managed bookkeeping. The facility director advocated for and managed resources, leading both formal and informal processes

Within the commune, the mayor was responsible for proposing a resource allocation plan for the entire commune, which covered environmental health services and other needs, following the formal resource allocation process. Due to limited available funding at the commune level, the mayor often sought additional funding from higher-level government bodies, NGOs, and UN agencies in coordination and with approval from the commune council.

It’s clear the mayor will be obliged each time to make pleas… The budget from the town hall, the taxes of our moms or our dads cannot make the town hall work. The mayor has to go out every time, go do advocacy, go do research, to get funding.… The mayor works with the council. The advice they gave to the mayor, the mayor executes. Because the mayor alone if he says he will do it, he will create problems myself

– Mayor

The health district management team, which involved actors such as the health district manager and the communication officers, coordinated monthly disbursements of supplies to healthcare facilities. Other departmental bureaucrats were not deeply involved in financial decisions, as they lacked budgetary control. Their primary role involved attending resource planning meetings to provide technical input. For instance, the hydraulics department relied on regional funding and did not engage in financial decision-making at the commune and health levels but could advise on the needs of various water infrastructure systems and their associated costs.

#### Non-governmental actors.

Non-governmental actors engaged in resource allocation comprised three main groups: villagers, community committees, and the diaspora (villagers who had relocated to larger cities or abroad, along with their descendants, but maintained connections with their home communities). Villagers supported environmental health services by providing their own water, paying water or health services fees, and providing in-kind support to construct, maintain, and clean healthcare facilities. Villagers’ efforts were coordinated by committees (e.g., WASH committees, COGES, and COSAN). COGES acted as the community’s liaison with healthcare facilities. They collaborated with other community-level committees and coordinated with healthcare facility directors and town halls. Their responsibilities encompassed the oversight of healthcare facility infrastructure, collaboration with health district bureaucrats, and facilitation of sustainable healthcare services, particularly for addressing non-cost-intensive needs.

We don’t go to the village to see someone to say that’s what’s going on. But we call on COGES, which is the representative of the community.

– Facility director

The diaspora were sometimes solicited by COGES and various government levels for funding to cover shortfalls. These solicitations were sometimes—but not always—successful.

Study participants mentioned that they targeted UN agencies (e.g., United Nations Development Program and UNICEF), international NGOs (e.g., World Vision, Plan International, Save the Children), and local NGOs with advocacy to request funds for environmental health services. When these advocacy efforts were successful, these actors contributed to funding environmental health infrastructure, regular supplies, maintenance, and human resources capacity building.

These non-governmental actors were highly valued for bridging the funding gap from higher government levels, especially for expensive infrastructure. However, their contributions were limited due to a need to align with their programmatic focus and internal guidelines. Their contributions were also often time-bound (e.g., consumables like soaps and alcohol gels were provided only for the duration of a specific project). For infrastructure projects, healthcare facilities frequently depended on NGOs for maintenance, with projects typically providing support for approximately one year post-construction. Larger international NGOs often sub-contracted with local individuals or companies to provide these services. This introduced complexities when subcontractors failed to meet expectations, requiring healthcare facilities or local governments to contact the NGO to follow up with sub-contractors. As one health district official described:

[The mayor] informed the contractor, who did not come for more than a week. We had to call on [the NGO]. [The NGO] contacted the contractor, who then came three days later.

– District communications officer

### Context

We identified five contextual domains that were relevant influences on resource allocation: political, legal, economic, sociocultural, and epidemiological. Our codebook also contained codes for geographic and ethical domains of the Context and Implementation of Complex Interventions framework, but we did not identify any strong relevant influences for these domains.

#### Political.

Despite the involvement of various stakeholders, political figures such as mayors and council members ultimately held budgetary authority. Politics was often perceived as a barrier, where decisions were made not necessarily based on needs but instead on political power.

Now even the constructions there are politicized. As long as you are at the top, you will not have a problem.

– Mayor.

We observed differences in the priority given to environmental health services versus the decision-making power of different actors involved in formal resource allocation. Healthcare facility workers and health district bureaucrats worked directly with environmental health services (e.g., WASH officers, hydraulics department personnel) and had observed and experienced the effects of environmental conditions first-hand. These actors were more likely to prioritized improving environmental health services. Many regarded water as fundamental to life, stating “water is life.” However, these actors had limited authority in the decision-making hierarchy. In contrast, most mayors, who possessed significant power in budget allocations, tended to focus more on broader health issues beyond environmental health services. Their focus included medical supplies and essential infrastructure, like delivery rooms, observation rooms, maternity wards, and the need for healthcare professionals, such as midwives and gynecologists.

We already have water. We’re not going to recommend water yet, but we have to ask for what we don’t have… [We] chose to build observation rooms, which are also part of the environmental conditions…. I take for example at the time of malaria, the fact of putting 10 people or 15 people in a single observation room is not good…. If the sick come, they can be hospitalized….

– Mayor

In informal resource allocation, NGOs held significant decision-making power due to their role as funders. However, NGOs’ internal program objectives were sometimes misaligned with local governments’ needs and priorities. Additional challenges included project cycles that funded upfront investment and a fixed time period of maintenance (often one year), without steady support for long-term operations and maintenance. These dynamic sometimes led to healthcare facilities receiving resources for services they did not consider urgent or some facilities feeling neglected.

If there is a lack of funding sometimes, we can search for funding from left to right. Are there NGOs, donors who will come? We can say that there is no [funding] in certain areas. They are disadvantaged. Even if it is not their area [of need], [healthcare facilities] accept but sometimes with difficulty, because each financial backer comes with his zone of intervention.

- Hydraulics district director

#### Legal.

Our interview guide specifically asked healthcare facility participants to describe policies related to environmental health resource allocation and provide written copies where available. However, knowledge and documentation related to specific policies was limited. Only one participant mentioned a concrete goal related to environmental health services, which was for healthcare facilities to have their water supply by 2023. No healthcare facility was able to produce budgets or other financial documents that included environmental health services, though some healthcare facilities did mention that documentation may have existed at higher levels of the health system but was not available at the day of the study visit.

Health district and departmental-level officials who were knowledgeable about policy mandates relevant to their roles were more inclined to focus on environmental health. However, the final decision-making authority in the resource allocation process rested with commune officials, including the mayor and council members. Although these elected officials were aware of the policies concerning environmental health services in healthcare facilities, they often could not detail the policy mandates related to their roles. As such, we observed a noteworthy lack of influence of environmental health policies on resource allocation at the commune level.

#### Economic.

The government lacked sufficient funding and human resources to achieve and maintain environmental health services across all healthcare facilities. This shortfall in resources was not unique to the environmental health sector. Other areas of healthcare, such as general human resources, observation rooms, maternity wards, and supplies (e.g., blood pressure monitors and medications) were also underfunded.

The overall high costs of environmental services influenced resource allocation decisions. Participants recognized water as a crucial element of environmental health, but water infrastructure was often funded last due to its high costs. The expenses associated with water infrastructure could vary dramatically, ranging from ten to thirty times the cost of constructing two blocks of latrines, depending on their size and the water source’s location.

#### Socio-cultural.

Most participants highlighted the significant role of in-kind donations from the community for environmental health services. Strong social ties among the diaspora were also important for larger cash donations that contributed to funding high-cost projects. The local community and diaspora’s support reflected a strong volunteerism and communal cooperation culture. However, the voluntary nature and limited budget posed challenges. One participant noted that, because community members volunteered, their contributions were made whenever they wished, which was not necessarily consistent or available when needed:

Someone who does volunteer work, you are not always going to force him to do it…. It is not easy. If he comes today, he may not come tomorrow.

- District Communications officer.

### Epidemiological

Epidemiologic factors both strengthened and hindered resource allocation for environmental health services. Endemic diseases and other health conditions competed for priority. Malaria was frequently mentioned as requiring more resources for medication and patient observation. Participants also expressed concerns about vaccine-preventable diseases and ensuring that vaccination campaigns were adequately resourced.

However, disease outbreaks perceived to be related to environmental health also boosted attention and available resources. For example, participants cited the cholera pandemic to stress the importance of environmental health services’ quantity and quality to prevent disease. The COVID-19 pandemic had also resulted in an influx of resources. However, these resources were often geared towards short-term response, such as donations of soap or alcohol-based handrub. One nurse described that COVID-19 support was waning and that healthcare workers were expecting to purchase supplies out of pocket to cope with shortages.

When there are COVID campaigns, they bring us a lot of soap and bibs. But currently we don’t even have bibs and soap. Maybe around the 17th [of the month] there will be a COVID campaign? They will surely bring us soaps, gels and bibs, too. That’s the only thing that doesn’t last… Currently we don’t use soap here. It’s only with alcohol-based gel that we work. [As COVID support is ending], if there is no more opportunity to have bleach as before, we pay them with our money. It is with our money that we buy the soaps.

- Nurse

## Discussion

We explored resource allocation for environmental health services in rural Niger’s healthcare facilities through qualitative interviews with 11 local government officials, 16 healthcare facility workers, and six community leaders. Niger has similarities to other decentralized systems in low- and middle-income countries, particularly in rural Africa [31–33,37,38], and offers insight into how actors navigate the challenges of providing services in resource-limited environments.

We found that formal resource allocation encompassed annual budgets and monthly supplies, while informal mechanisms depended on healthcare workers’ out-of-pocket expenses, unpaid labor, community support, water revenue, and contributions from NGOs, UN agencies, and the diaspora. Informal resource allocation was critical to fill the gap when formal processes were slow or insufficient to meet needs. Government actors within formal frameworks were limited by their roles but often advocated for additional informal funds. Key factors influencing resource allocation included epidemiological, legal, socio-cultural, political, and economic considerations, with notable politicization of budgets at the commune level.

### Actors navigate a complex, fluid resource allocation processes

We found that most actors engaged in both formal and informal resource allocation processes. Formal processes were respected and followed but acknowledged as insufficient to meet needs—either due to lack of resources, delays, or frequently both. When formal processes fell short, actors turned to informal processes to meet their needs. Formal and informal processes were not seen as contradictory or conflicting but essential to work together to provide services.

Our findings contradict two stereotypes regarding resource allocation and financing for environmental health services. The first is that underfunding is attributable to a lack of political will [39,40]. We found that many different actors—including elected government officials—took considerable effort above and beyond their official duties to attempt to secure resources through informal processes. Niger is a highly resource constrained context, and these efforts were not always successful, so actors balanced a complex set of health and non-health needs within their communities—sometimes investing in other priorities besides environmental health. However, we found that participants in our sample perceived environmental health services to be high value and wanted to improve them. Monitoring data from the WHO and UNICEF suggest that this finding may apply in other countries. The past five years have seen considerable increases in the number of countries that have adopted national standards, guidelines, and roadmaps for environmental health services in healthcare facilities [41], with commitments reinvigorated through a 2024 *Global Framework for Action* which outlines specific national actions to accelerate progress [42].

Understanding the capacity and incentives of local actors offers a clearer view of their incentives and limitations in resource allocation, rather than simply attributing issues to a lack of political will [43,44]. It also has implications for targeting effective advocacy messages and other approaches to influence policies for resource allocation. For example, if officials targeted for advocacy already believe in the importance of environmental health services, merely sensitizing them to the benefits is likely insufficient. Instead, recognizing the local context of fluid formal and informal resource allocations, support should be provided to enable actors to better manage and optimize various resources. This may be achieved by assessing the cost-effectiveness of investments in environmental health services and evaluating the trade-offs made between different funding priorities.

The second stereotype that our findings contradict is that government bureaucracy is a rigid structure, which can sometimes hinder service delivery [45]. Rather, our findings depict government actors as flexible in informal processes. For instance, healthcare facilities actors sought resources from community committees and affluent individuals, while health district and department bureaucrats engaged NGOs and the diaspora. Moreover, elected officials like mayors interacted with community committees like COGES, NGOs, and UN agencies. This fluid interaction within informal mechanisms allowed government actors to advocate for diverse support from non-government actors.

The flexibility allowed by local actors likely reflects a high degree of decentralization in Niger. Prior research in other countries highlights the critical role of local bureaucrats in implementing innovative policies [46] and local officials in bridging formal and informal mechanisms [47], particularly within decentralized systems. Financing that combines resources from local and national government, NGOs, and bilateral and multilateral agencies for environmental health services is common in low- and middle-income countries [48]. We therefore suspect that other low-income countries with this type of blended financing model would also share a similar complex blend of formal and informal resource allocation processes.

### Power and knowledge imbalances influence resource allocation

In rural Niger, both government actors and community management committees advocated for resources from entities that they perceived as more powerful and resourceful than themselves. Government officials held considerable power over formal resource allocation. However, government officials held more limited power over informal resource allocation, where NGOs and other external donors had stronger influence to dictate where and how funds were spent—sometimes in ways that were not fully aligned with the government’s perceived needs and priorities.

Unsurprisingly, political power influenced resource allocation. While environmental health services were prioritized, and local actors took considerable effort to secure resources, those resources were sometimes not distributed efficiently or equitably. Examples of inefficiency include funding for projects that were not well aligned the needs and priorities of local healthcare workers and administrators and allocations of resources to areas based on political alliances rather than needs. Efficient resource allocation is particularly critical in Niger and other low-resource settings to maximize the benefits of already scare resources. We observed two challenges that contributed to inefficiency.

First, there is a disconnect between field expertise and decision-making power at healthcare facilities. Healthcare workers who are most knowledgeable about local needs and submitted plans and requests to local officials, but decisions were ultimately made by elected officials with little health expertise. Furthermore, large investments were typically funded by actors outside the community. The extent to which outside actors consulted community members varied, and in some instances resulted in investments that were better aligned with donor rather than local priorities.

The disconnect between field expertise and decision-making power is not unique to Niger and has been documented elsewhere [21]. Studies of spending on environmental health services in other countries with highly decentralized health systems and strong local autonomy have found similarly, recommending capacity building of local governments to enhance skills related to budgeting and financial planning [23]. In the short term, including local actors in project proposal development is essential to ensure that the project addresses local needs and incorporates sustainability after completion. In the long run, external donors should commit to long-term initiatives that promote democratic decision-making, balancing flexibility with accountability, establishing communication channels between field experts and decision-makers [49], and enhancing local experts’ capacity to deliver high-quality evidence. Long-term, they could advocate for governance frameworks that mandate evidence-based decision-making [50].

The second challenge we observed contributing to inefficient resource allocation is the weak influence of legal and policy frameworks at the local level, in part due to politicized decision-making. At the commune level, the mayor and council members, elected through a political process, hold primary power over resource allocation. Local governments play a vital role in interpreting national targets while representing local needs [51,52]. However, we found that participants in our sample frequently knew that national targets existed but were unable to describe specifics.

Disconnect between the local and national levels in Niger may in part reflect political instability and weak national government in Niger. Some communities in our sample had limited or sporadic contact with regional or national authorities due to security concerns that at times restricted travel and communications. We expect that other countries in the Sahel region with a history of political instability and health systems that share similar structural characteristics may show a similar disconnect between the local and national levels [53], whereas least-developed countries that are more politically stable may see greater influence of national-level policies. Misalignment between national objectives and local achievements, typical in decentralized systems, can slow progress toward national targets. Communication and advocacy are essential to address this but are likely to prove challenging in the current politically turbulent Nigerien context. Public policy frameworks suggest that policy solutions must be prepared and advocated so that they can be adopted when the political climate is favorable [54]. Donors aiming to reduce power and knowledge imbalances in resource allocation by democratizing decision-making processes should acknowledge the capacities and incentives of local actors. This approach provides a clearer understanding of their motivations and constraints in resource allocation, rather than merely attributing challenges to a lack of political will [42,43]. Moreover, donors should begin by democratizing their own resource allocation processes when working with local actors.

### Informal processes are enablers and challenges for effective and sustainable resource allocation

In our study in rural Niger, informal resource allocation processes emerged as vital enablers, allowing healthcare facilities to provide environmental health services despite limited government resources. This scenario is common in rural, resource-limited contexts [8,55]. There is little evidence from healthcare facility programs, but evidence from community-based programs is mixed. Reliance on uncompensated community volunteers—particularly as a substitute for professionalized services—has faced sustainability critiques [56,57]. However, financing schemes that allow community members to pay for services with in-kind contributions such as labor or goods (e.g., livestock) have improve sustainability of community-based water systems in other African countries [58]. Remittances from the diaspora are a critical source of funding for development, exceeding official development aid in absolute amount and contributing substantially to development, including for healthcare and WASH services [59,60]. Participants in our sample identified water vending from healthcare facility water towers as a source of revenue, which has been trialed previously with some initial success but challenges with sustainability [61]. Overall, how these informal processes adapt and scale across healthcare facilities is largely unknown.

In contexts where government funding is severely limited, relying on informal mechanisms may be the only viable option to ensure the availability of services. These mechanisms allow actors to quickly mobilize resources in response to immediate needs, demonstrating flexibility and adaptability in contrast to the rigidity of formal allocation timelines and bureaucratic procedures [62]. In formal settings, government actors are often confined to their designated roles and authority, limiting their ability to respond swiftly to emerging challenges. However, there are drawbacks to informal resource allocation mechanisms. Notably, they raise equity concerns, where healthcare works and patience face increased fees or in-kind contributions to compensate for lack of adequate formal resourcing.

Ultimately, frameworks and recommendations from the WHO and UNICEF for country action to reach universal access to environmental health services in healthcare facilities recommend strong, government-led efforts to develop, fund, and implement costed plans [63]. Yet pragmatically, adequate resourcing for environmental health services in healthcare facilities exclusively through formal processes is not realistic in the short or medium term for Niger or many other low- and middle-income countries. In the interim, future research can aid this transition by identifying resource allocation processes that ensure services are still provided without potentially undermining the long-term effort of government system strengthening.

Our study offers a snapshot of how formal and informal processes operate together in Niger, but these dynamics will likely change over time in different stages of development or in different political or health systems. Further study should acknowledge this reality by considering different economic development and political contexts when exploring solutions that do not undermine the long term goal of health systems strengthening.

### Limitations

We conducted this study in healthcare facilities receiving an NGO program, and as such participant responses may have been biased either to emphasize effectiveness or to highlight severe conditions for more aid. While using an independent consulting firm for data collection reduces potential bias, some reporting bias may remain. Our study area also likely represents greater resource availability in Niger than other areas that lack strong NGO partnerships.

We sampled local actors, predominantly at the commune levels. We found that these participants were minimally aware of resource allocation processes and reported minimal influence of policies at the regional and national levels on their decision making. However, we did not sample provincial or national level actors to triangulate these findings, which would strengthen future research.

### Conclusion

Our study provides insight into how formal and informal processes for resource allocation in Niger work together in a complex, flexible arrangement. Formal resource allocation met some but not all needs, leading actors to rely on informal sources such as community support, water sales, increased healthcare service fees, out-of-pocket costs to healthcare workers, and contributions from NGOs, UN entities, and the diaspora. Achieving sustainable and efficient resource allocation remains challenging due to a disconnect between field expertise and decision-making, exacerbated by politicization.

Informal resource allocation processes emerged as vital enablers, allowing healthcare facilities to provide services despite limited government resources. These processes were an important stopgap to ensure service delivery, but how they contribute to—or undermine—the long-term goal of sustainable, professional service provision is unclear.

## Supplementary Material

SI file 1 **S1 Data**. Facility demographic data.

SI file 2 **S1 Table**. Codebook.

## Figures and Tables

**Table 1.** Processes, actors, and context constructs from the policy triangle framework, which are theorized to influence resource allocation for environmental health services in healthcare facilities, adapted from the Walt and Gilson policy triangle framework [24].

Construct	Definition
Processes	Procedures for allocating financial and human resources for environmental health services in healthcare facilities
Actors	Stakeholders involved in the resource allocation process, distinguished by their distinct roles (i.e., a specific set of expectations and responsibilities that are associated with a particular position or function)
Contexts	Unique combination of characteristics and conditions in which the resource allocation process is situated and operates

https://doi.org/10.1371/journal.pwat.0000330.t001

**Table 2.** Characteristics of healthcare facilities in the program area.

Healthcare facility characteristic	Sample size
Water source	
Onsite borehole or tubewell	17 (50%)
Offsite borehole or tubewell	9 (26%)
Dug well	5 (15%)
Delivered by cart or tanker	2 (6%)
Offsite piped supply	1 (3%)
Facility has a water tower	7 (21%)
Facility tests water for microbial contamination	0 (0%)
Facility has at least one improved toilet available	30 (88%)
Method of sharps waste disposal	
Burning in a dedicated incinerator or burner	17 (50%)
Open burning in an unprotected area	8 (25%)
Open burning in a protected pit	5 (15%)
Transported and disposed offsite	4(12%)
Facility always has soap available for handwashing	15 (44%)
Facility always has gloves available for patient care	24 (71%)

https://doi.org/10.1371/journal.pwat.0000330.t002

**Table 3.** Demographic characteristics of interview participants.

Demographic characteristic	Sample size
*Gender*	
Male	26 (79%)
Female	7 (21%)
*Job title*	
Healthcare facility participants	
President or Vice president - COGES	7 (21%)
Facility Nurse	6 (18%)
Facility Director or Deputy Director	4 (12%)
Facility Preceptor	4 (12%)
Facility Head nurse	1 (3%)
Government participants	
Mayor or Deputy Mayor	5 (15%)
Departmental director of hydraulics and sanitation	2 (6%)
District Communications officer	2 (6%)
Health district manager	1 (3%)
District WASH officer	1 (3%)
*Years in current position*	
0–2 years	12 (36%)
3–5 years	8 (24%)
6–10 years	7 (21%)
>10 years	6 (18%)

https://doi.org/10.1371/journal.pwat.0000330.t003

## Data Availability

Data are not publicly available to protect the privacy of research subjects. These are small healthcare facilities in Niger with a limited number of healthcare workers and government officials. Given the limited number of health facilities in rural Niger as well as the rich contextual clues in the transcripts about their job duties, work environment, and local geography, making the data publicly available poses a risk that study participants may be identified. Please use the following point of contact from the University of North Carolina at Chapel Hill’s Institutional Review Board for questions or concerns regarding participant confidentiality and data availability: irb_questions@unc.edu.
