# Supplementary material for: Resource allocation for environmental health services in healthcare facilities: A qualitative case study from Niger
Source: PLOS Water. Author manuscript; Available in PMC 2025 Sep 23. (PMC12453085; doi:10.1371/journal.pwat.0000330)
Supplement: SI file 2 — S1 Table. Codebook. [file NIHMS2111173-supplement-SI_file_2.docx]

S1 Table: Codebook

| Theme | Definition |
| --- | --- |
| 1. Resource allocation process | Procedure for allocating resources for environmental health service in healthcare facilities, for financial and human resources |
| 1.1.Formal process | Government resource allocation procedures for both monetary resources and government-paid personnel, which are typically aligned with the government timeline |
| 1.2.Informal process | Resource allocation from entities outside of government, such as community funding, volunteerism, and contributions from civil societies, which is not bound by the strict timelines of government resource allocation. |
| 2. Actors | Actors are stakeholders involved in the resource allocation distinguished by their distinct roles (i.e., a specific set of expectations and responsibilities that are associated with a particular position or function) |
| 2.1.Government actors | Individuals who operate within the governmental system, including elected officials (such as mayors and council members), district and departmental bureaucrats, and healthcare workers |
| 2.2.Non-government actors | Entities outside the government system who also play significant roles in the allocation of resources for environmental health services in healthcare. These entities include villagers, community committees, local and international Non-Governmental Organizations, United Nations agencies, and private individuals. |
| 3. Context | Unique combination of characteristics and conditions in which the resource allocation process is situated and operates |
| 3.1 Political | The prioritization of issues by different actors in contrast to their involvement and power in the decision-making process |
| 3.2 Legal | Government actors' understanding of the policy content and mandate in guiding resource allocation prioritization |
| 3.3 Economic | The availability of resources within and outside the government system encompassing both monetary and non-monetary resources. |
| 3.4 Socio-cultural | Beliefs and social norms of the population that might influence resource allocation for environmental health services in healthcare facilities |
| 3.5. Epidemiological | Actors' perceptions of diseases and their burdens, which can influence the prioritization of resources. |
| 3.6 Geographical | Characteristics of the physical environment, such as the location of water sources and water availability. |
